# Hospitalization Costs of Respiratory Diseases Attributable to Temperature in Australia and Projections for Future Costs in the 2030s and 2050s under Climate Change

**DOI:** 10.3390/ijerph19159706

**Published:** 2022-08-06

**Authors:** Michael Tong, Berhanu Wondmagegn, Jianjun Xiang, Alana Hansen, Keith Dear, Dino Pisaniello, Blesson Varghese, Jianguo Xiao, Le Jian, Benjamin Scalley, Monika Nitschke, John Nairn, Hilary Bambrick, Jonathan Karnon, Peng Bi

**Affiliations:** 1 School of Public Health, The University of Adelaide, Adelaide, SA 5005, Australia; 2 Department of Health, Government of Western Australia, Perth, WA 6004, Australia; 3 Department of Health, Government of South Australia, Adelaide, SA 5000, Australia; 4 Australian Bureau of Meteorology, Adelaide, SA 5000, Australia; 5 School of Public Health and Social Work, Queensland University of Technology, Brisbane, QL 4000, Australia; 6 College of Medicine and Public Health, Flinders University, Bedford Park, SA 5001, Australia

**Keywords:** hospitalization cost, respiratory diseases, temperature, climate change

## Abstract

This study aimed to estimate respiratory disease hospitalization costs attributable to ambient temperatures and to estimate the future hospitalization costs in Australia. The associations between daily hospitalization costs for respiratory diseases and temperatures in Sydney and Perth over the study period of 2010–2016 were analyzed using distributed non-linear lag models. Future hospitalization costs were estimated based on three predicted climate change scenarios-RCP2.6, RCP4.5 and RCP8.5. The estimated respiratory disease hospitalization costs attributable to ambient temperatures increased from 493.2 million Australian dollars (AUD) in the 2010s to more than AUD 700 million in 2050s in Sydney and from AUD 98.0 million to about AUD 150 million in Perth. The current cold attributable fraction in Sydney (23.7%) and Perth (11.2%) is estimated to decline by the middle of this century to (18.1–20.1%) and (5.1–6.6%), respectively, while the heat-attributable fraction for respiratory disease is expected to gradually increase from 2.6% up to 5.5% in Perth. Limitations of this study should be noted, such as lacking information on individual-level exposures, local air pollution levels, and other behavioral risks, which is common in such ecological studies. Nonetheless, this study found both cold and hot temperatures increased the overall hospitalization costs for respiratory diseases, although the attributable fractions varied. The largest contributor was cold temperatures. While respiratory disease hospitalization costs will increase in the future, climate change may result in a decrease in the cold attributable fraction and an increase in the heat attributable fraction, depending on the location.

## 1. Introduction

Global temperature has risen markedly by 1 °C since industrialization began around 1750, largely due to human activities and the increases in greenhouse gas emissions, e.g., from energy production, transportation, agriculture and industries [1,2]. With increasing temperatures, climate change is one of the greatest threats to human health in the 21st century [3,4]. The impact of climate change and its impact on population health has been explored extensively over the past two decades [5]. Many epidemiological studies have demonstrated the negative impacts of climate change on population health [6], and growing evidence has shown increasing temperatures were associated with increased morbidity and mortality of a range of temperature-sensitive diseases, including direct heat-associated diseases, e.g., heatstroke, cardiovascular diseases, renal diseases, mental health disorders, and indirect vector-borne infectious diseases, e.g., dengue fever, malaria, Ross River virus, and hemorrhagic fever with renal syndrome [6,7,8,9,10,11,12,13,14,15]. However, it should be noted that cold temperatures can also be associated with poor health outcomes, and cold-related diseases should not be overlooked because of the recent focus on heat-related diseases in the context of climate change. One of the typical disease categories that is associated with cold temperatures is respiratory disease [16,17]. Studies across the world in different climatic conditions and geographical zones have almost reached a consensus that respiratory diseases are more likely to occur during the winter period or in low-temperature conditions [16,17,18,19]. Nevertheless, the associated economic burden on the healthcare system has not been well explored. Only several studies explored the impact of climate change on healthcare costs in Australia, and most of them focused on the heat-related diseases and heat effects on healthcare costs [20,21,22,23,24]. Climate change has added the urgency to better understand the economic burden of diseases not only due to increasing temperatures but also possible reduced cold effects on healthcare costs due to temperature increase.

Studies found that both hot and cold temperature exposure can increase the risk of respiratory diseases [17,25,26]. A study in the USA indicated each degree above a threshold of the daily mean temperature of 28.9 °C was associated with a 2.7–3.1% increase in same-day hospitalizations due to respiratory diseases [25]. In Europe, a study conducted among 12 cities found 1 °C increase in daily maximum temperature was associated with a 1.2–2.1% increase in respiratory admissions within a lag of 0–3 days [26]. By contrast, a study in Finland found 1 °C decrease in average temperature increased the risks for upper respiratory tract infections by 4.3% and lower respiratory tract infections by 2.1% [17]. Moreover, studies in Australia and China showed both hot and cold temperatures increase the risk of respiratory diseases [18,19], and several other studies also indicated that a large temperature change significantly increased the risk of respiratory diseases [27,28]. Although previous studies found a significant association between ambient temperature exposures and respiratory diseases, very limited studies have assessed the healthcare costs attributable to ambient temperatures nor estimated the future healthcare costs for respiratory diseases in the context of climate change [29].

Australia has had a universal health care scheme (Medicare) since 1984, which covers all costs of public hospital services. The Australian state and territory governments cover about 68% of the overall cost of health care in Australia; the other 32% of health care costs are shared by individuals (17%), private health insurers (9%) and non-government organizations (6%) [30]. In Australia, it is predicted that the annual average temperature will increase by 0.6–1.3 °C in the 2030s above the reference period of 1986–2005 and by up to 2.8–5.1 °C at the end of the century [31]. Such temperature change could further impact the incidence of respiratory diseases and increase or decrease the associated economic burden on healthcare in this region.

The present study aimed to examine respiratory disease hospitalization costs attributable to non-optimum ambient temperature, quantify the attributable fractions from hot and cold temperatures and estimate the associated future hospitalization costs in two Australian cities—Sydney and Perth in the context of climate change. The results will help local and regional government, health authorities and communities to have a better understanding of climate change attributable hospitalization costs; plan, evaluate and optimize current climate change adaptation strategies; and better direct medical resources to respond to respiratory diseases in the context of climate change.

## 2. Materials and Methods

### 2.1. Study Setting

This study was conducted in two cities in Australia—Sydney and Perth. Sydney is located on Australia’s east coast and has a humid subtropical climate with warm summers and cool winters (humid subtropical Koppen climate zone) [32]. It is the largest city in Australia, with a population of 5.5 million in 2020, living in an area of 12,368 square kilometers [33]. Perth, about 4000 km away from Sydney, is located on Australia’s west coast and has a Mediterranean climate with hot and dry summers and cool and wet winters (Mediterranean Koppen climate zone) [32]. It is the fourth largest city in Australia, with a population of 2.1 million in 2020, living in a metropolitan area of 6418 square kilometers [33]. These cities were selected to evaluate the respiratory disease hospitalization costs in different demographic and climatic contexts in Australia [34]. Three time periods were used in this study: 2010s, 2030s and 2050s. The baseline period of July 2010–June 2016 was defined as the 2010s; the corresponding future periods 2030–2036 and 2050–5056 were defined as 2030s and 2050s, respectively.

### 2.2. Data Sources

#### 2.2.1. Hospitalisation Cost Data

Respiratory disease hospitalization costs: Daily respiratory disease (ICD-10-AM: J00-J99) hospitalization costs from public hospitals over the study period from July 2010 to June 2016 in Sydney and Perth were obtained from the Australian National Independent Hospital Pricing Authority (IHPA). The daily respiratory disease hospitalization costs were aggregated by the date of admission for statistical analysis. The patient hospitalization costs included all costs, including prescription medications, treatment and medical examination for respiratory health conditions incurred during the hospitalization period.

#### 2.2.2. Meteorological Data

Meteorological data: Daily minimum (Tmin) and maximum (Tmax) temperatures were obtained from the Australian Bureau of Meteorology. Daily mean (Tmean) temperature was calculated by the average of Tmin and Tmax. Tmin, Tmean and Tmax are the averages calculated from 17 weather observation stations in Sydney and 13 weather observation stations in Perth (Figure S1). Daily mean temperatures for the 2030s and 2050s were projected based on three Commonwealth Scientific and Industrial Research Organisation (CSIRO) —defined Representative Concentration Pathways (RCPs). Depending on the RCP emission scenarios, the temperature in Sydney is projected to increase by 0.9–1.2 °C for the 2030s, relative to the reference period 1986–2005, and by 1.0–2.0 °C for the 2050s (Table S1) [35]. In Perth, the temperature is projected to increase 0.8–1.0 °C by the 2030s and by 0.9–1.8 °C for the 2050s, relative to the reference period 1986–2005 [35].

In addition, population data for the baseline period 2010s and projection periods 2030s and 2050s were obtained from the Australian Bureau of Statistics (ABS) for the two cities [33]. For the projection periods, we adopted the ABS ‘medium’ levels of fertility, life expectancy, net overseas migration and interstate flows for the two cities. Daily populations were estimated using the linear interpolation method [36].

### 2.3. Statistical Analyses

The methods for statistical analyses were described in our previous studies on assessing the effects of temperature on healthcare costs in Adelaide, Perth and Sydney [20,21,22]. Two-stage data analyses were performed to explore the association between temperature and hospitalization costs and predict future hospitalization costs. In the first stage, the relationships between daily mean temperature and daily respiratory disease hospitalization costs were estimated using a generalized linear time series regression with a distributed lag non-linear model (DLNM) [37]. In the second stage, the future temperature-attributable respiratory disease hospitalization costs were estimated based on the baseline associations, projected temperatures, and populations for the 2030s and 2050s.

#### 2.3.1. First Stage

In order to assess the shape of the exposure–lag–response relationship, a DLNM model with gamma distribution was fitted simultaneously to estimate the possible non-linear relationship and lagged effects of temperature on hospitalization costs [37,38,39]. The model controlled for long-term trends, seasonality, weekday variations and public holidays. The exposure–response curves were modeled using a natural cubic spline for temperature with three internal knots placed at the 10th, 75th, and 90th percentiles of temperature distributions, and the lag–response curves with a natural cubic spline with an intercept and three internal knots placed at equally spaced values in the log scale [40,41]. In order to control for location-specific seasonality and long-term trends, a B-spline with 8 degrees of freedom (*df*) per year for time [bs(time, 8*df* per year × 6 years)] in Sydney and 4*df* per year for time [bs(time, 4*df* per year × 6 years)] in Perth were included in our models [39]. In order to control for weekday variations, the day of the week (dow) was also included in the model. Public holidays (pubhol) were controlled by using a binary variable. The temperature effects were calculated relative to the optimum temperature (i.e., the temperature at which the minimum relative risk for hospitalization costs occurred), which was obtained from the cumulative exposure–response curve for respiratory disease hospitalization costs, as per the methods of Gasparrini et al. [38,42]. The location-specific models were described as follows:

$$\begin{array}{l} \mathrm{In}\;\mathrm{Sydney}:\;\log\left[E\left(Y_{t}\right)\right]\\ \;=\alpha \;+cb\left(Temp_{t}\right)+bs\left(time,\;8df\;per\;year\times 6\;years\right)\\ \;+dow_{t}+pubhol_{t} \end{array}$$

$$\begin{array}{l} \mathrm{In}\;\mathrm{Perth}:\;\log\left[E\left(Y_{t}\right)\right]\\ \;=\alpha \;+cb\left(Temp_{t}\right)+bs\left(time,4df\;per\;year\times 6\;years\right)\\ \;+dow_{t}+pubhol_{t} \end{array}$$

where *Y_t_* is respiratory disease hospitalization costs on day *t*; α is the intercept; *cb(Tempt)* is the cross-basis natural cubic spline function for daily mean temperature with both response and lag dimension applied from the DLNM; *bs(time, 8 and 4df per year × 6 years)* is the B-spline with degrees of freedom per year multiplied by the 6 year study period, adjusted for seasonality and long-term trend in Sydney and Perth [43]; *time* is in days; *dow* is the day of the week on day *t*; and *pubhol* is a binary variable representing public holidays on day *t.*

The optimum temperature for respiratory disease hospitalization costs was identified via an overall cumulative exposure–response curve in the first stage, and the outcome below or above the optimum temperature was assigned as the temperature effect due to exposures [37,39]. The daily mean temperature was chosen as the index providing the best fit in the analysis of respiratory disease hospitalization costs. In order to completely capture the temperature-related hospitalization costs, a maximum of 14 lag days was used in the model, and the value shows the best fit for the respiratory diseases in this study. We tested these modeling choices in sensitivity analysis, which was conducted by changing temperature metrics from daily mean to minimum and maximum temperatures, *df* for time from 4*df* to 8*df* per year, and the maximum lag days of 14 days to 7–21 days, to compare and best capture the effects of temperature on hospitalization costs. Residual analysis and autocorrelation tests were conducted to evaluate the goodness of model fit and autocorrelation. In order to ensure the hospitalization costs were comparable across the different years, consumer price index (CPI) data were obtained from the Australian Bureau of Statistics [44], and the daily hospitalization costs were adjusted for inflation and standardized to the second quarter of 2016 in Australian dollars (AUD).

#### 2.3.2. Second Stage

After the baseline exposure–response relationships between daily temperatures and hospitalization costs were obtained from the first stage analysis, future respiratory disease hospitalization costs were estimated based on the baseline model and projected temperature changes in the 2030s and 2050s. The future temperature effects on hospitalization costs were estimated under three temperature scenarios (RCP2.6, RCP4.5, and RCP8.5 emission scenarios) for the periods 2030s and 2050s. Hospitalization costs and fractions attributable to temperature with reference to the optimum temperature of minimum relative risk were calculated to show temperature attributable costs (*AC*) and attributable fractions (*AF*). The *AC* and *AF* were calculated using the method of Gasparrini and Leone [40]. The *AC* and *AF* are defined as:

$$AC_{x,t}=AF_{x,t}\times C_{t}$$

$$AF_{x,t}=1-exp\left(-\sum_{l=0}^{L}\beta_{x_{t-l},\;l}\right)$$

where *x* is the daily mean temperature exposure on day *t*; *C_t_* is the daily hospitalization costs on day *t*. $\beta_{x_{t-l},\;l}$ is the natural logarithm of RR given exposure on day *t − l* after *l* days have elapsed. The effects of projected daily mean temperatures above the current observed range were estimated using Monte Carlo simulation (*n* = 1000) [40,45]. Estimated hospitalization costs were also adjusted for future population estimates in the 2030s and 2050s as per ABS projections [33].

All statistical analyses were performed using R software (R Foundation for Statistical Computing, Vienna, Austria) with the packages “dlnm”, “tsModel” and function “attrdl” [40,46].

## 3. Results

### 3.1. Descriptive Results of Daily Temperatures and Hospitalization Costs

**Figure 1:**
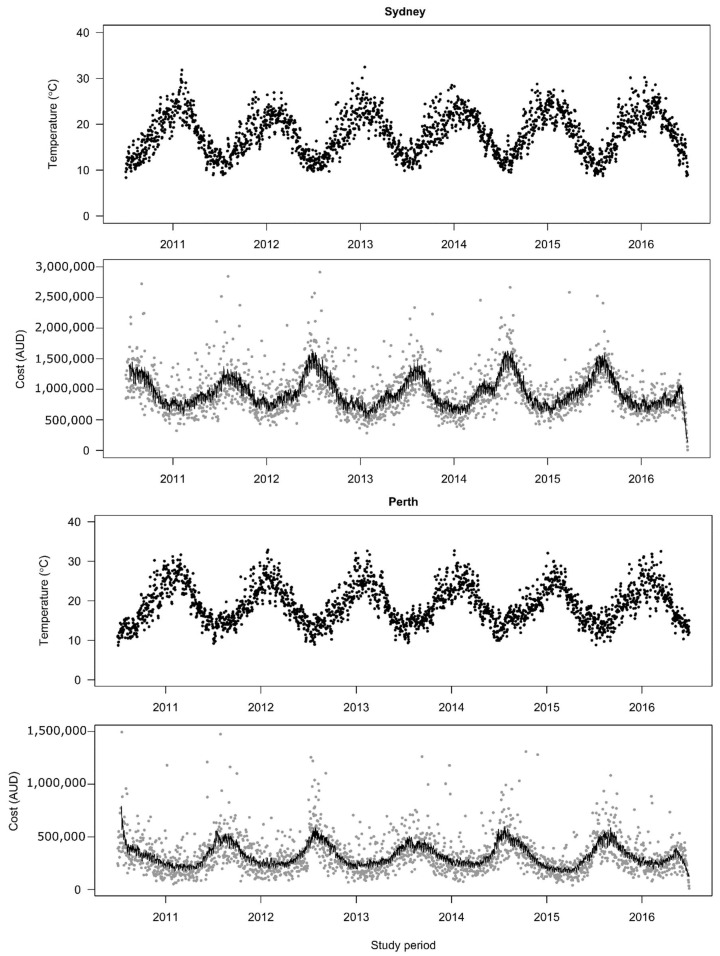
Time-series plots for daily mean temperatures and daily hospitalization costs of respiratory diseases in Sydney and Perth, 2010–2016. Costs are significantly correlated with the temperatures (Spearman rho = −0.49, *p* < 0.001) in Sydney and (Spearman rho = −0.43, *p* < 0.001) in Perth. Dark points are daily mean temperatures; Grey points are daily hospitalization costs; Black line is the best fit line generated based on daily hospitalization costs.

Table 1 shows the daily temperatures and respiratory disease hospitalization costs in Sydney and Perth for the baseline study period. The daily mean temperatures in the two cities are quite similar, with 18.1 °C (SD: 4.7) in Sydney and 18.9 °C (SD: 5.0) in Perth over the study period of 2010–2016. In Sydney, there were AUD 2080.3 million in hospitalization costs for respiratory disease; in Perth, there were AUD 709.3 million in hospitalization costs. Figure 1 shows a time series plot of daily mean temperatures and respiratory disease hospitalization costs in Sydney and Perth over the study period, which may indicate an inverse relationship between daily mean temperatures and respiratory disease hospitalization costs.

### 3.2. Exposure–Response Relationship between Daily Mean Temperatures and Hospitalization Costs

**Figure 2:**
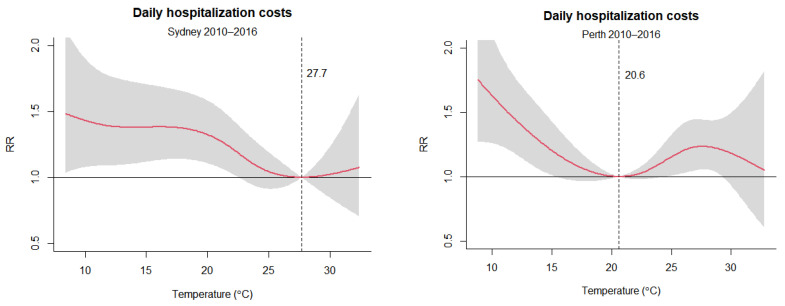
Overall cumulative exposure–response relationships between daily mean temperatures and daily hospitalization costs for 14 lag days in Sydney and Perth, 2010–2016. RR is the relative risk for hospitalization costs. The optimum temperatures are 27.7 °C and 20.6 °C for respiratory disease hospitalization costs in Sydney and Perth, respectively. The red lines represent the cumulative relative risk, and the grey shaded areas represent the 95% confidence interval.

Figure 2 shows the overall cumulative exposure–response curves for daily mean temperatures and respiratory disease hospitalization costs in Sydney and Perth. The relationships between temperatures and respiratory disease hospitalization costs are presented as non-linear associations. The effects of temperature on respiratory disease hospitalization costs were more obvious at low temperatures than at high temperatures in both cities, which is consistent with the implied relationship seen in Figure 1. Specifically, the respiratory disease hospitalization costs slowly increase as temperatures decrease below the optimum temperature in Sydney, while there is a steeper increase in the respiratory disease hospitalization costs at lower temperatures in Perth, despite different optimum temperatures identified in the two cities. By contrast, only in Perth do temperatures above the optimum show noteworthy effects on hospitalization costs for respiratory diseases.

**Figure 3:**
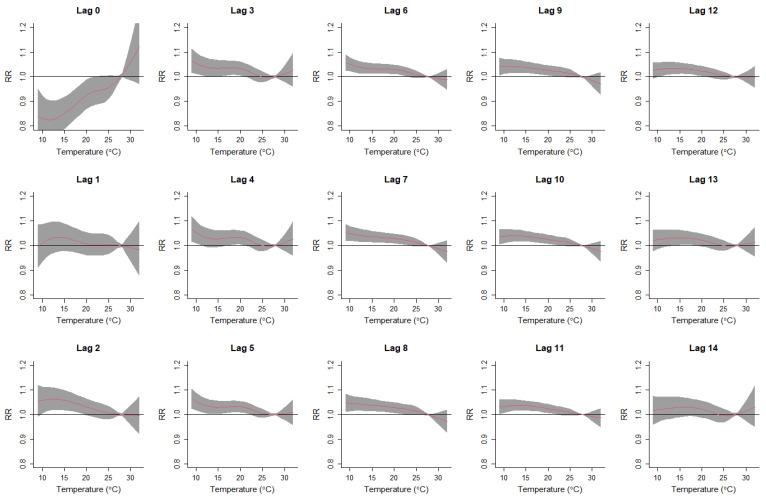
Lag-specific exposure–response curves for daily mean temperatures and respiratory disease hospitalization costs in Sydney, 2010–2016. RR is relative risk for respiratory disease hospitalization costs. The red lines represent the lag-specific relative risk, and the grey shaded areas represent the 95% confidence interval.

**Figure 4:**
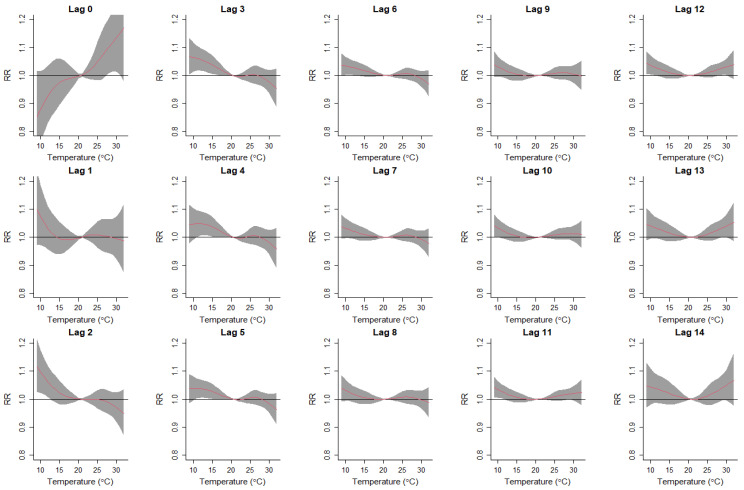
Lag-specific exposure–response curves for daily mean temperatures and respiratory disease hospitalization costs in Perth, 2010–2016. RR is relative risk for respiratory disease hospitalization costs. The red lines represent the lag-specific relative risk, and the grey shaded areas represent the 95% confidence interval.

Figure 3 and Figure 4 further present the lag-specific exposure–response curves of daily mean temperatures and respiratory disease hospitalization costs in Sydney and Perth. In Sydney, on days of low-temperature exposure (Lag 0 days), respiratory hospitalization costs were reduced relative to the optimum temperature. However, increased costs due to the lagged effects of low temperatures were evident from Lag 2–12 days. Exposure to higher temperatures in Sydney did not present an effect on the hospitalization costs. In Perth, lagged exposures to low temperatures (Lag 2–12 days) showed a similar pattern as in Sydney, with increased hospitalization costs for respiratory diseases at lower temperatures. In addition, exposure to high temperatures (Lag 0 day) increased hospitalization costs in Perth.

### 3.3. The Effects of Current and Future Temperature Increase on Hospitalization Costs for Respiratory Diseases

Table 2 reports the estimates of the total fraction of hospitalization costs for respiratory diseases due to temperature during the baseline study period of the 2010s. Overall, 23.71% (95% CI: 8.39–34.96) and 13.82% (95% CI: 5.95–20.90) of respiratory disease hospitalization costs were attributed to non-optimum temperatures in Sydney and Perth, respectively, in the 2010s. The total fraction can be separated into two components due to cold and hot temperatures. The comparison of the two components shows that cold temperatures are responsible for most respiratory disease hospitalization costs with 23.69% (95% CI: 8.28–34.99) and 11.20% (95% CI: 2.93–18.67), compared to 0.02% (95% CI: −0.08–0.11) and 2.64% (95% CI: 0.65–4.72) for hot temperatures in Sydney and Perth, respectively. Despite the larger component attributable to temperatures in Sydney, the contribution from hot temperatures is noteworthy in Perth only. The results are consistent with the overall cumulative exposure–response curves in Figure 2.

For the estimation of future hospitalization costs for respiratory diseases under three RCP scenarios (RCP2.6, RCP4.5 and RCP8.5) over the periods of 2030s and 2050s (Table 2), the estimated overall fraction attributable to a temperature slightly reduced from 23.71% in 2010s to 20.26%, 20.07% and 19.67% under the RCP2.6, RCP4.5 and RCP8.5 scenarios, respectively, for 2030s in Sydney. The estimates would be further reduced to 18.09% under high emission scenario RCP8.5 in the 2050s. A similar pattern was evident for Perth, with the overall fraction attributable to temperature reducing from 13.82% in the 2010s to 11.34–11.20% in the 2030s to 11.24–10.54% in the 2050s. Specifically, the estimated cold-attributable fraction of hospitalization costs for respiratory diseases would reduce from 20.24–19.64% in the 2030s to 20.08–18.04% in the 2050s in Sydney, and the cold-attributable fraction would reduce from 6.75–6.46% in the 2030s to 6.63–5.07% in the 2050s in Perth. By contrast, the heat-attributable fraction of hospitalization costs for respiratory diseases in Perth would slightly increase from 4.61–4.76% in the 2030s to 4.63–5.48% in the 2050s under the three different climate change scenarios, but in Sydney, there is no substantial increase in the heat-attributable fraction. Nevertheless, it should be noted that the absolute costs attributed to temperature increased substantially from AUD 493.2 million in the 2010s to more than AUD 700 million in the 2050s in Sydney and from AUD 98.0 million to around AUD 150 million during the same period in Perth.

The results of the sensitivity analyses can be found in Supplementary Materials. These analyses include changing the daily mean temperatures to a daily minimum and maximum temperatures to capture the association between daily temperatures and hospitalization costs for respiratory diseases (Figure S2), the *df* of the B-spline for the calendar year from 4 to 8 per year (Figure S3), maximum lag days from 7 to 21 days (Figure S4), and residuals for the DLNM models (Figure S5). The residuals for the DLNM models followed a normal distribution, and no significant autocorrelations were found in the residuals.

## 4. Discussion

This study presented results on the effects of daily mean temperature on hospitalization costs for respiratory diseases in two Australian cities with different climatic characteristics and estimated the changes in such costs under different climate change scenarios in the future. We found that Sydney has higher hospitalization costs (AUD 2080.3 million) for respiratory diseases than Perth (AUD 709.3 million), which cannot be entirely explained by the population size of the two cities (5.5 million and 2.1 million, respectively). This could be due to Sydney being a larger multicultural city with a higher number of communities of new Australians, who might not be able to develop their adaptation behaviors to the Australian climate, and higher numbers of people without or not willing to use air conditioners due to electricity costs [47,48,49], which could lead to potential bias for results. Furthermore, the high-density population in Sydney may facilitate the occurrence or transmission of certain types of respiratory diseases, e.g., flu and pneumonia [27,50].

We found there were overall non-linear relationships between ambient temperature exposures and respiratory disease hospitalization costs in Sydney and Perth. Both cold and hot temperatures can increase hospitalization costs, with a higher risk at lower temperatures in both cities. This is consistent with other studies exploring the impact of temperature on morbidity due to respiratory diseases, suggesting more cold-related respiratory diseases than heat-related respiratory diseases [18,19,27,51]. Furthermore, the lag0 day exposure to cold is associated with reduced costs for respiratory disease hospitalizations in Sydney. This could be explained by limited human outdoor activities during sudden cold exposure and hence reduced cold exposure and cold-related respiratory disease hospitalization costs, or patients may seek medical care from the emergency department for acute onset conditions such as asthma that were not accounted for hospitalization costs. However, exposure to cold temperatures from 2 days to 12 days is associated with an increase in hospitalization costs for respiratory diseases. Previous studies also showed the delayed cold effect on respiratory diseases, which could persist beyond 2 weeks [52,53]. In Perth, the trend with reduced hospitalization costs on lag0 day is also apparent, although it was not obvious.

We found an acute heat effect on hospitalization costs on the day of heat exposure in Perth but not in Sydney. The observed differences between the two cities could be due to the different climates and population adaptation. The respiratory disease optimum temperature identified in Sydney, which has a higher relative humidity of 60–70% across the year [54], was 27.7 °C, and this optimum temperature is plausible for people living in higher relative humid areas as the higher temperature is required for evaporation of perspiration. In Perth, which has a dry climate with a relative humidity of 40–50% across the year [55], the optimum temperature was 20.6 °C. Humans may adapt to the temperate climate with warm to hot summers but be more susceptible to cold temperatures or a hot and dry environment. Nevertheless, the two optimum temperatures are generally in accordance with those reported in other studies [41,56]. The integrated effect of these factors may finally result in the difference in the heat effect on respiratory disease hospitalization costs between Sydney and Perth.

The study estimated AUD 493.2 million and AUD 98.0 million in hospitalization costs for respiratory diseases associated with ambient temperature exposures over the baseline study period in Sydney and Perth, respectively. The cold attributable fraction was as high as 23.7% in Sydney, compared with 11.2% in Perth. The results for both cities indicated that most of the hospitalization costs for respiratory diseases were attributed to cold temperatures. The possible explanatory mechanism could be that cold temperatures are more likely to cause pathophysiological responses that affect the immune system and increase the susceptibility to respiratory diseases, e.g., pneumonia caused by viruses and bacteria kills more people than other respiratory diseases [16,17,57,58,59,60]. Further, cold weather may lead to more wood-fired heaters in homes and generate particulate matter in enclosed environments, which can be linked to respiratory conditions [61]. Other reasons could include that people may stay indoors and crowd together during wintertime, facilitate virus spread or bacteria transmission and increase the incidence of disease [58]. These would, in turn, increase the economic burden on healthcare due to respiratory diseases when the temperature is lower. By contrast, the heat attributable fractions in the two cities were much smaller. The comparison between cold and heat attributable fractions for hospitalization costs indicates that the burden of respiratory disease due to heat in Sydney and Perth is lower relative to the burden during the cold winter period, which is consistent with other studies [18,19,27,53].

The estimation of the future hospitalization costs attributable to temperature for respiratory diseases showed a substantial increase from about AUD 500 million and AUD 100 million at the baseline period in Sydney and Perth, respectively, to approximately AUD 700 million and AUD 150 million by midcentury, despite its declining attributable fractions to non-optimum temperature. The possible explanation could be the future burgeoning population by the middle of this century, with up to 9.1 million population in Sydney and 3.7 million population in Perth [33]. In addition, the increasing aging population in Australia would also be more susceptible to respiratory diseases [58,62]. Thus, the absolute hospitalization costs will be increased substantially. Furthermore, it is worthy to note that the heat-attributable fraction for respiratory diseases was estimated to double in Perth from 2.64% in the 2010s up to 5.48% in the 2050s. This indicates that close attention should be paid to heat-related respiratory diseases in the future. In the context of climate change, more extreme weather events such as heatwaves, bushfires, droughts, dust storms, longer pollen seasons and air pollution would further deteriorate respiratory health and conditions, such as asthma, respiratory tract infections and chronic obstructive pulmonary disease, and increase the associated hospitalization costs in the future [63,64]. Climate change adaptation measures such as greater air conditioner uptake, use of health heat warning systems, and community awareness improvement/behavior change could decrease the risk in the context of climate change.

This study is the first to examine the temperature effects on hospitalization costs for respiratory diseases over a long time period in Australia. We employed a DLNM model to control for confounding effects, including seasonality, long-term trends, day-of-week and public holidays. We estimated both cold and hot temperature attributable hospitalization costs for respiratory diseases in cities with different climates and demographics. We also projected such hospitalization costs in the future in the context of climate change. In addition, the comparison between temperature effects in Sydney and Perth may suggest an acclimatization effect. The observed heterogeneity of the temperature effect on hospitalization costs for respiratory diseases may also indicate a need to conduct location-specific and disease-specific studies to explore the temperature effect on population health outcomes in the future.

Several limitations of this study should be acknowledged. First, we assumed the baseline exposure–response relationships for the estimation of future respiratory disease hospitalization costs would not change under different climate change scenarios in the future. However, human behavioral adaptations to changing climate in the long term may play a certain role in mitigating the effect of temperature on respiratory diseases. This may change the impact of temperature changes on respiratory disease hospitalization costs. Second, this study did not consider individual-level exposures, personal indoor and outdoor daily activity patterns, local air pollution levels and other behavioral risks because of the data availability issue, which may alter the temperature effect on hospitalization costs for respiratory diseases. Furthermore, technological progress and innovation may impact the emission of greenhouse gases, which could make a difference in such projection. Third, the hospitalization costs for the respiratory disease may reflect only severe and acute episodes, which may underestimate the overall burden, including those who attended the emergency department, and costs due to lost productivity for days off work. Fourth, the hospitalization costs were adjusted based on the consumer price index up to the second quarter of 2016, which may not reflect the future healthcare costs due to economic fluctuations, including inflation, deflation or discounted cash flow. Fifth, age could affect respiratory health risks and hospitalization costs. However, due to restricted data availability, only aggregated daily respiratory disease data were obtained. Lastly, the respiratory disease hospitalization costs were gathered from two cities in Australia, which may limit the generalizability of research findings to other areas and regions. Subsequent studies to explore costs in sub-tropical and inland locations would be warranted. In addition, respiratory disease hospitalization costs due to emerging infectious diseases and pandemics such as COVID-19, avian flu, SARS, and MERS in the future may not be particularly temperature-sensitive but cannot be projected.

## 5. Conclusions

Cold temperatures are associated with increased hospitalization costs for respiratory diseases with delayed effects in Sydney and Perth and are responsible for most of the temperature-attributable economic burden of respiratory disease hospitalizations. The cold attributable fraction is likely to decline due to global warming from the current 23.7% to 18% and 11.2% to 5.1% by midcentury in Sydney and Perth, respectively. Additionally, the hospitalization costs for respiratory diseases attributed to heat are estimated to gradually increase in the future. The estimated overall hospitalization costs attributable to temperature will increase from currently about AUD 500 million to AUD 700 million in Sydney and AUD 100 million to AUD 150 million in Perth by the middle of this century. Interpretation of the results should be made with caution as other factors such as individual-level exposures, local air pollution levels, and other behavioral risks were not considered. Nonetheless, the findings highlight the significant impact of cold temperatures on the economic burden of respiratory diseases currently in Australia and also may suggest that overall temperature attributable respiratory disease hospitalization costs are likely to increase substantially in Australia in the context of climate change.

## Figures and Tables

**Table 1:** Descriptive statistics of daily mean temperature and respiratory disease hospitalization cost in Sydney and Perth, 2010–2016.

Characteristics	Sydney	Perth
Daily mean temperature °C (SD)	18.1 (4.7)	18.9 (5.0)
Hospitalization costs	AUD 2080.3 million	AUD 709.3 million
Observation days	2192	2192
Population	5.5 million	2.1 million
Population median age	36.3 years	37.1 years
Climate	Temperate climate	Mediterranean climate

SD: Standard Deviation. AUD: Australian dollars.

**Table 2:** Total fraction (%) of hospitalization costs for respiratory diseases attributable to temperature, reported as overall, cold and heat components with 95% confidence intervals (CI).

		Sydney				Perth			
Period		Costs (Million AUD)	Overall (95% CI)	Cold (95% CI)	Heat (95% CI)	Costs (Million AUD)	Overall (95% CI)	Cold (95% CI)	Heat (95% CI)
2010s									
	Baseline	493.2	**23.71** (8.39; 34.96)	**23.69** (8.28; 34.99)	0.02 (−0.08; 0.11)	98.0	**13.82** (5.95; 20.90)	**11.20** (2.93; 18.67)	**2.64** (0.65; 4.72)
2030s									
	RCP2.6	623.8	**20.26** (6.02; 30.73)	**20.24** (5.84; 30.97)	0.03 (−0.24; 0.28)	118.6	**11.34** (5.79; 16.69)	**6.75** (1.22; 11.80)	**4.61** (1.10; 8.25)
	RCP4.5	617.7	**20.07** (5.92; 30.53)	**20.04** (5.73; 30.79)	0.03 (−0.25; 0.29)	118.1	**11.29** (5.79; 16.62)	**6.64** (1.16; 11.64)	**4.67** (1.10; 8.35)
	RCP8.5	605.4	**19.67** (5.73; 30.12)	**19.64** (5.49; 30.32)	0.03 (−0.29; 0.34)	117.2	**11.20** (5.73; 16.40)	**6.46** (1.05; 11.38)	**4.76** (1.14; 8.50)
2050s									
	RCP2.6	784.5	**20.11** (5.96; 30.54)	**20.08** (5.77; 30.81)	0.03 (−0.25; 0.30)	155.9	**11.24** (5.76; 16.54)	**6.63** (1.16; 11.62)	**4.63** (1.09; 8.30)
	RCP4.5	753.2	**19.30** (5.58; 29.67)	**19.28** (5.20; 29.94)	0.04 (−0.33; 0.39)	151.2	**10.90** (5.54; 15.93)	**5.91** (0.80; 10.62)	**5.01** (1.25; 8.93)
	RCP8.5	705.8	**18.09** (5.06; 28.04)	**18.04** (4.62; 28.40)	0.06 (−0.49; 0.57)	146.1	**10.54** (5.40; 15.69)	**5.07** (0.37; 9.41)	**5.48** (1.39; 9.78)

Bold cells indicate temperature-attributable hospitalization costs.

## Data Availability

The datasets used and analyzed are available from the corresponding author on reasonable request and after approval by the Independent Hospital Pricing Authority.

## Supplementary Materials

The following supporting information can be downloaded at: https://www.mdpi.com/article/10.3390/ijerph19159706/s1, Figure S1: Study areas and weather stations in Sydney and Perth; Table S1: Future projected temperature increases in 2030s and 2050s; Figure S2: Pooled estimates of temperature on hospitalization costs when changing the daily temperatures in Sydney and Perth; Figure S3: Pooled estimates of temperature on hospitalization costs when changing the *df* for time in Sydney and Perth; Figure S4: Pooled estimates of temperature on hospitalization costs when changing the max lag period in Sydney and Perth; Figure S5: Histogram, Scatter plot, ACF and PACT plot of residuals derived from DLNM model.
