# Supplementary material for: Hospitalization Costs of Respiratory Diseases Attributable to Temperature in Australia and Projections for Future Costs in the 2030s and 2050s under Climate Change
Source: Int J Environ Res Public Health. 2022 Aug 6;19(15):9706. doi: 10.3390/ijerph19159706 (PMC9368165; doi:10.3390/ijerph19159706)
Supplement: Supplementary file 1 [file ijerph-19-09706-s001.zip › ijerph-1802579-supplementary.pdf]

Supplementary materials

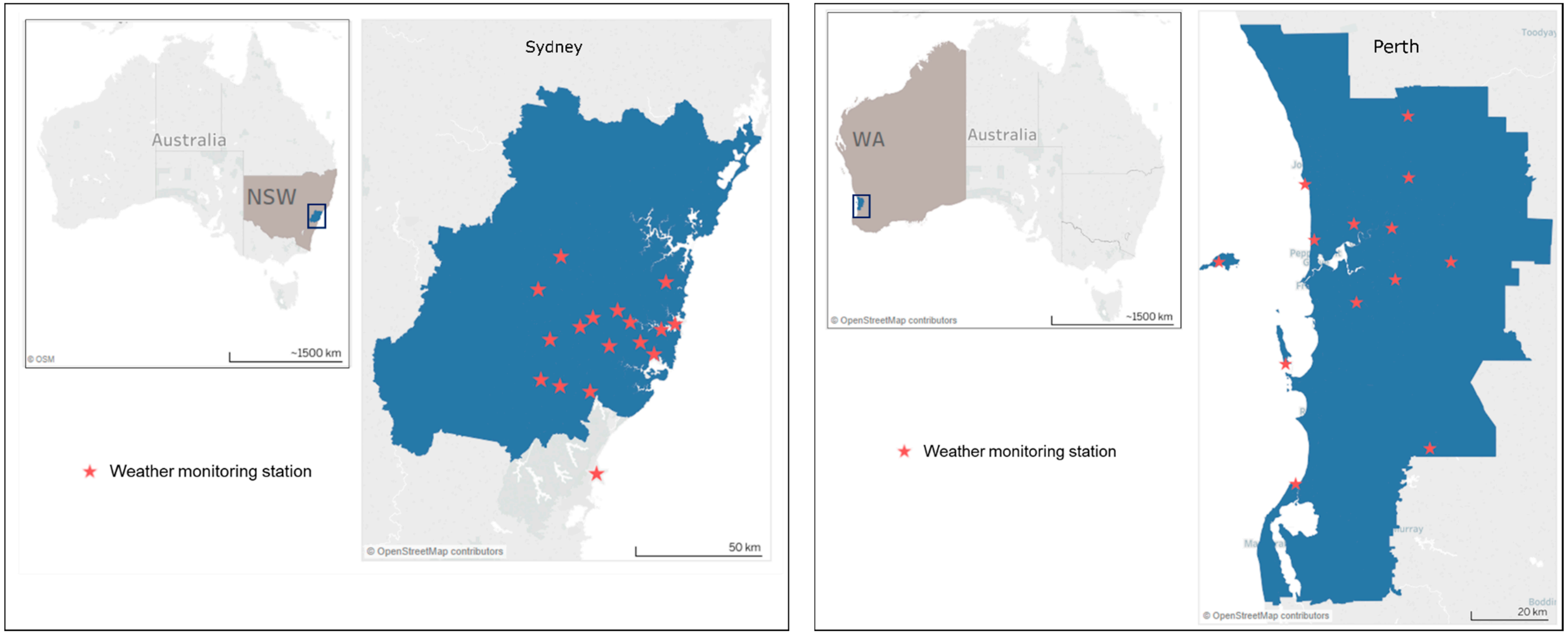

Figure S1. Study areas and weather stations in Sydney and Perth.

In Sydney, the 17 weather observation stations are Sydney Airport Amo (Station ID: 066037), Terrey Hills Aws (Station ID: 066059), Sydney (Observatory Hill) (Station ID: 066062), Parramatta North (Masons Drive) (Station ID: 066124), Bankstown Airport Aws (Station ID: 066137), Canterbury Racecourse Aws (Station ID: 066194), Sydney Harbour (Wedding Cake West) (Station ID: 066196), Sydney Olympic Park AWS (Archery Centre) (Station ID: 066212), Prospect Reservoir (Station ID: 067019), Richmond Raaf (Station ID: 067105), Badgerys Creek Aws (Station ID: 067108), Penrith (Station ID: 067113), Horsley Park Equestrian Centre Aws (Station ID: 067119), Camden Airport Aws (Station ID: 068192), Bellambi (Station ID: 068228), Campbelltown (Mount Annan) (Station ID: 068257), Holsworthy Defence Aws (Station ID: 068263).

In Perth, the 13 weather observation stations are Perth Metro (Station ID: 009225), Perth Airport (Station ID: 009021), Swanbourne (Station ID: 009215), Gosnells City (Station ID: 009106), Jandakot Aero (Station ID: 009172), Hillarys Boat Harbour (Station ID: 009265), Millendon (Station ID: 009281), Bickley (Station ID: 009240), Rottnest Island (Station ID: 009193), Pearce RAAF (Station ID: 009053), Garden Island HSF (Station ID: 009256), Karnet (Station ID: 009111), Mandurah (Station ID: 009977).

Table S1. Future projected temperature increases in 2030s and 2050s relative to the climate reference period (1986-2005), and mean temperatures for period 1986-2005 and 2010-2016 in Sydney and Perth, Australia.

Site	Sydney		Perth	
Time period	1986-2005	2010-2016	1986-2005	2010-2016
Mean temperature	17.76	18.07	18.08	18.91
	Projected temperature increases (SD)			
	Sydney		Perth	
Time period	2030s	2050s	2030s	2050s
RCP2.6	0.9 (0.2)	1.0 (0.3)	0.8 (0.2)*	0.9 (0.2)
RCP4.5	1.0 (0.2)	1.4 (0.4)	0.9 (0.2)	1.3 (0.3)
RCP8.5	1.2 (0.3)	2.0 (0.4)	1.0 (0.2)	1.8 (0.3)

*Temperature increase for estimation of future hospitalization costs during 2030s under RCP2.6 in Perth is added only 0.01 ℃ as it has reached the projected temperature increase during the baseline study period 2010-2016.

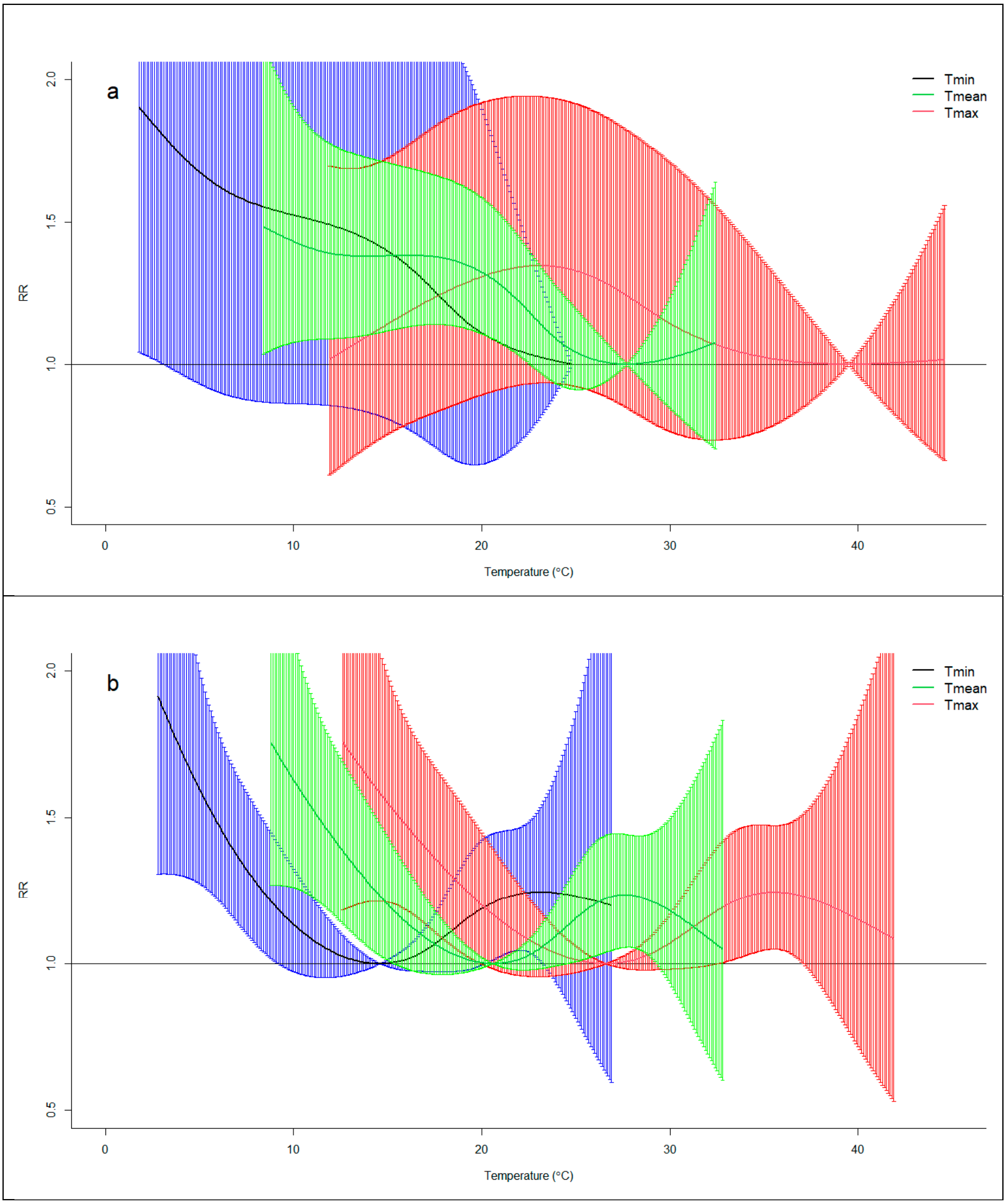

Figure S2. Pooled estimates of temperature on hospitalization costs when changing the daily temperatures in Sydney (a) and Perth (b), 2010-2016. RR is the relative risk for respiratory disease hospitalization costs.

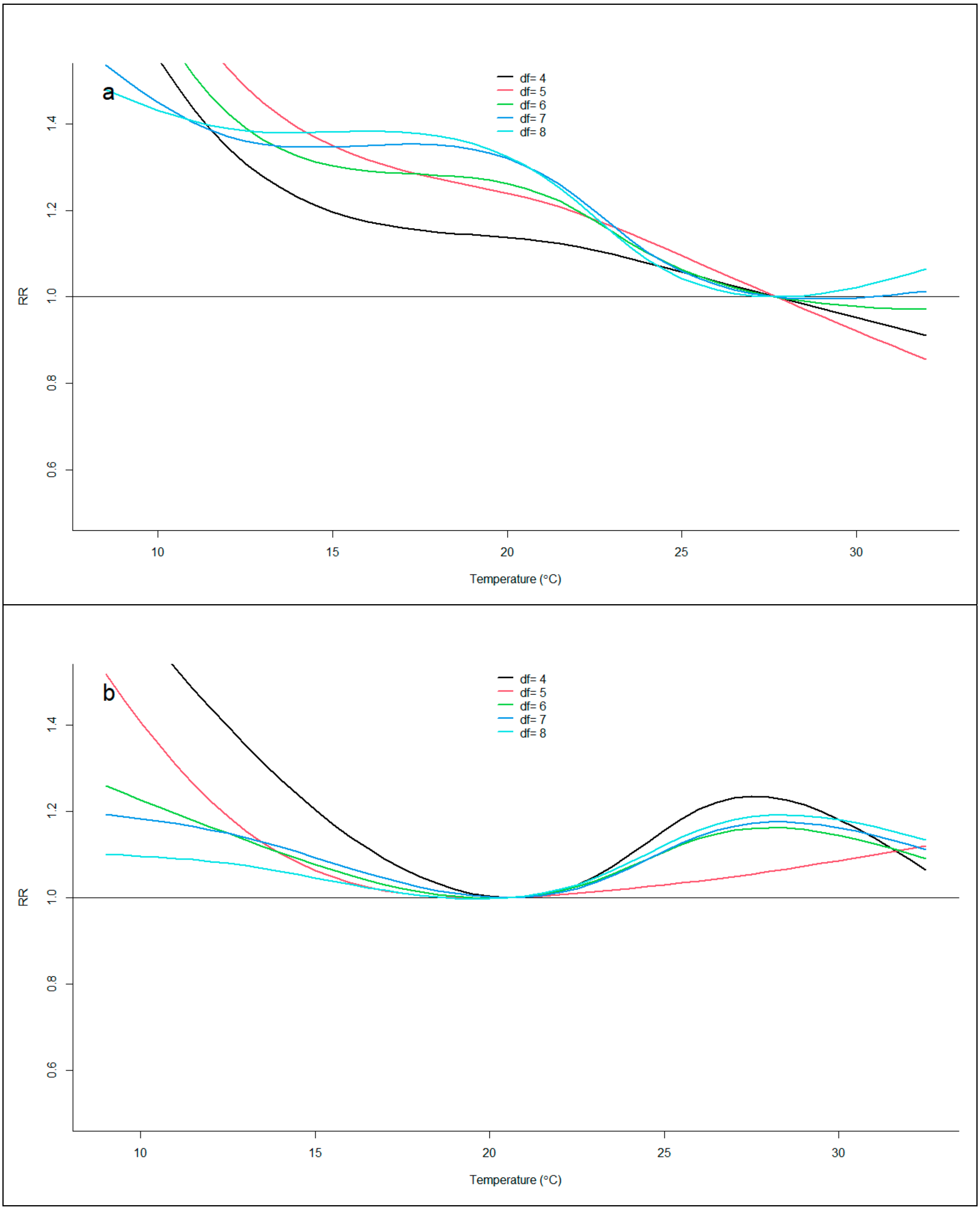

Figure S3. Pooled estimates of temperature on hospitalization costs when changing the df for time in Sydney (a) and Perth (b), 2010-2016. RR is the relative risk for respiratory disease hospitalization costs.

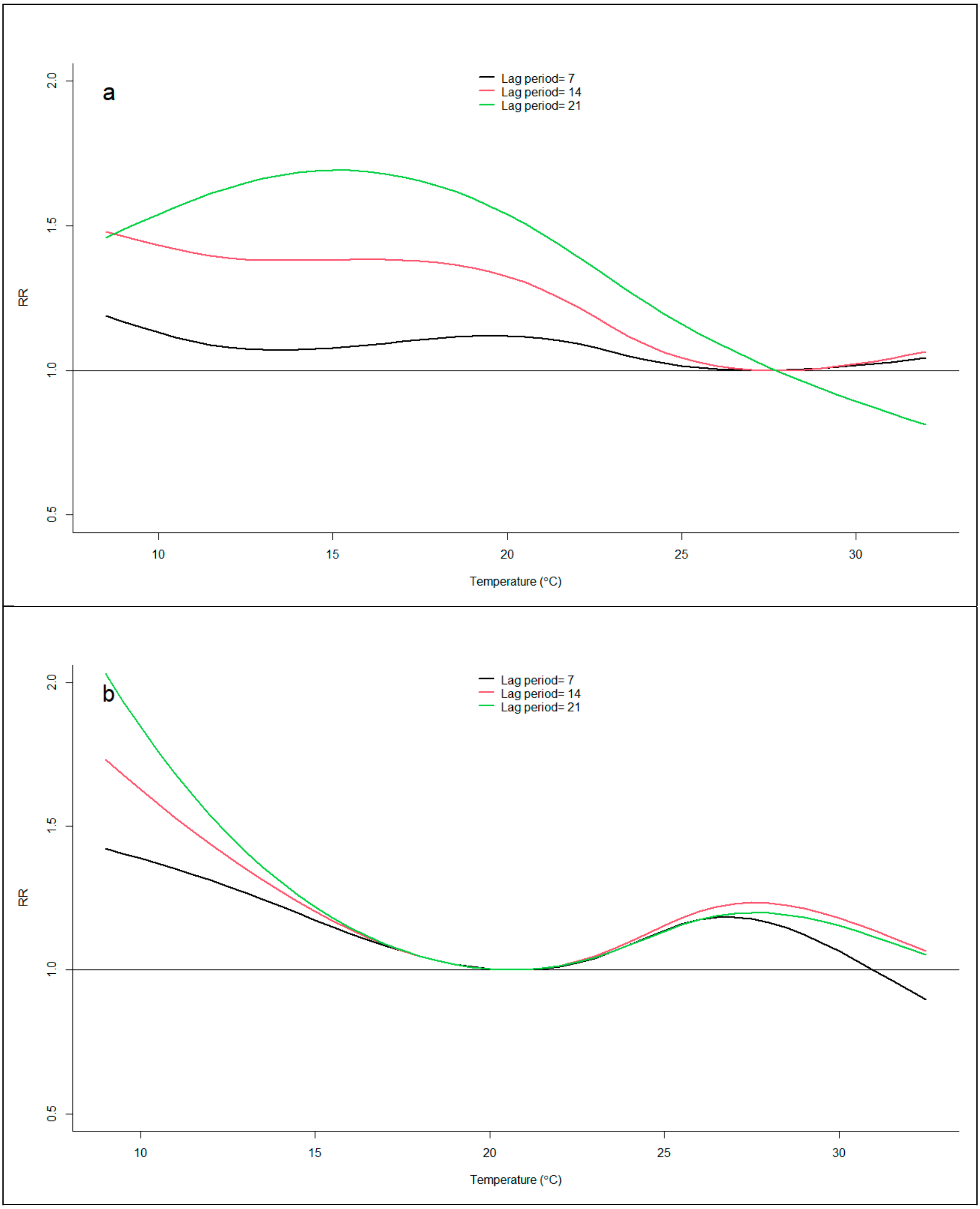

Figure S4. Pooled estimates of temperature on hospitalization costs when changing the max lag period in Sydney (a) and Perth (b), 2010-2016. RR is the relative risk for respiratory disease hospitalization costs.

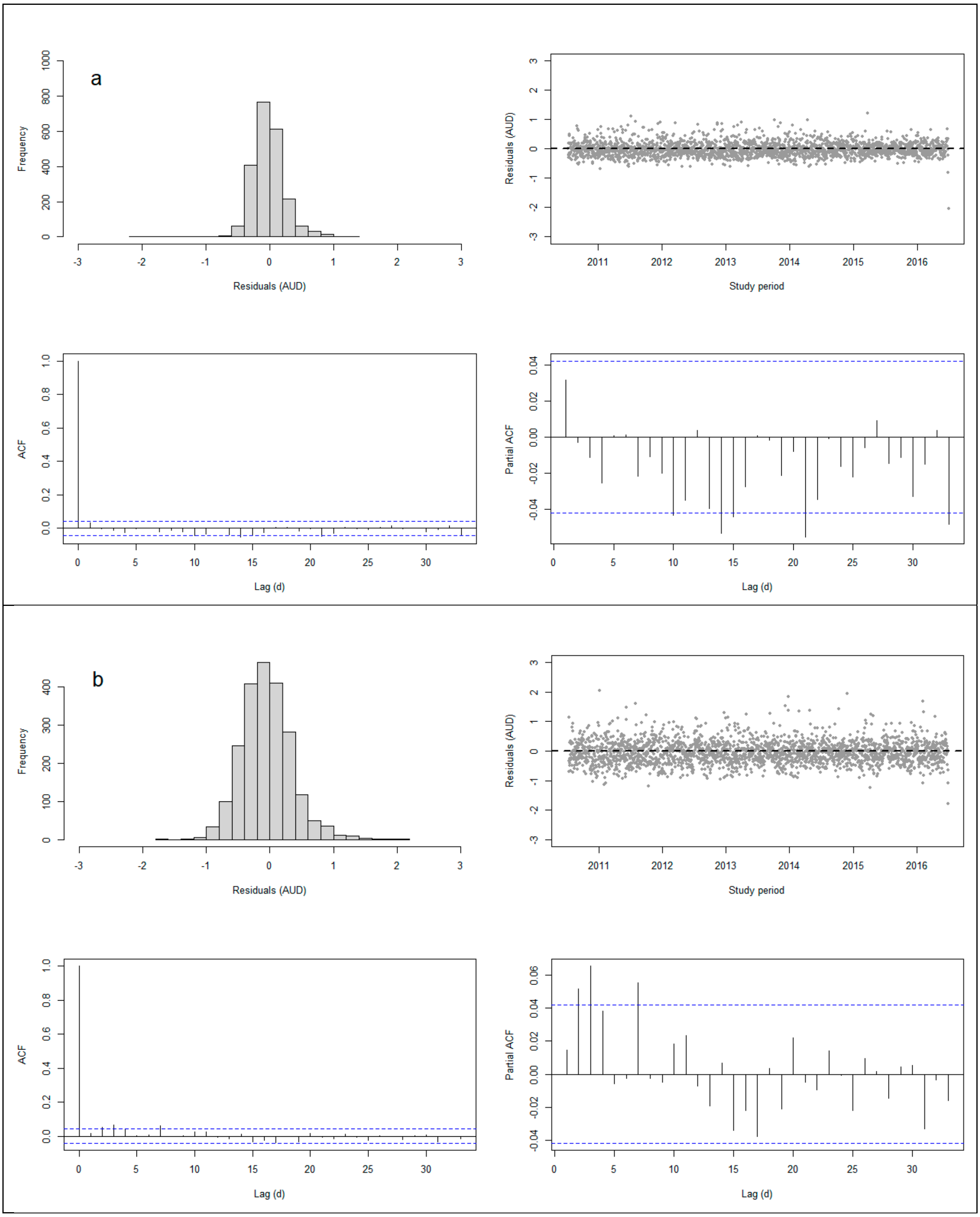

Figure S5. Histogram, Scatter plot, ACF (autocorrelation function) and PACF (partial autocorrelation function) plot of residuals derived from DLNM model for respiratory disease hospitalization costs in Sydney (a) and Perth (b), 2010-2016.
